# The association between postpartum hemorrhage and postpartum depression: A Swedish national register-based study

**DOI:** 10.1371/journal.pone.0255938

**Published:** 2021-08-11

**Authors:** Can Liu, Alexander Butwick, Anna Sand, Anna-Karin Wikström, Jonathan M. Snowden, Olof Stephansson

**Affiliations:** 1 Clinical Epidemiology Division, Department of Medicine, Solna, Karolinska Institutet, Stockholm, Sweden; 2 Department of Public Health Sciences, Stockholm University, Stockholm, Sweden; 3 Department of Anesthesiology, Perioperative, and Pain Medicine, Stanford University School of Medicine, Stanford, California, United States of America; 4 Department of Women’s Health, Karolinska University Hospital, Stockholm, Sweden; 5 Department of Women’s and Children’s Health, Uppsala University, Uppsala, Sweden; 6 School of Public Health, Oregon Health & Science University–Portland State University, Portland, Oregon, United States of America; Univesity of Iowa, UNITED STATES

## Abstract

**Background:**

Postpartum hemorrhage is an important cause of maternal death and morbidity. However, it is unclear whether women who experience postpartum hemorrhage are at an increased risk of postpartum depression.

**Objectives:**

To examine whether postpartum hemorrhage is associated with postpartum depression.

**Methods:**

We conducted a national register-based cohort study of 486,476 Swedish-born women who had a singleton livebirth between 2007 and 2014. We excluded women with pre-existing depression or who filled a prescription for an antidepressant before childbirth. We classified postpartum depression up to 12 months after giving birth by the presence of an International Classification of Diseases, version 10 (ICD-10) diagnosis code for depression or a filled outpatient prescription for an antidepressant. We used Cox proportional hazard models, adjusting for maternal sociodemographic and obstetric factors.

**Results:**

Postpartum depression was identified in 2.0% (630/31,663) of women with postpartum hemorrhage and 1.9% (8601/455,059) of women without postpartum hemorrhage. In our unadjusted analysis, postpartum hemorrhage was not associated with postpartum depression (unadjusted hazard ratio (HR) = 1.06, 95% confidence interval (CI) 0.97–1.15). After adjusting for maternal age, parity, education, cohabitation status, maternal smoking status, and early pregnancy maternal BMI, gestational age, and birthweight, the association did not appreciably change, with confidence intervals overlapping the null (adjusted HR = 1.08, 95% CI 0.99, 1.17).

**Conclusions:**

Within a population-based cohort of singleton women in Sweden with no prior history of depression, postpartum hemorrhage was not associated with postpartum depression.

## Introduction

Postpartum depression (PPD) is a major maternal health problem affecting 10–15% of women and birthing people after giving birth [1,2]. Known risk factors for PPD include a history of depression or anxiety [3], relationship dissatisfaction [4], exposure to domestic violence [5], lack of social support [6], stressful life events [4], maternal isolation [7], negative attitudes toward pregnancy [8], adverse neonatal outcomes such as preterm birth, small-for-gestational-age, low Apgar score and a major fetal congenital anomaly [9]. Less clear is whether women who experience postpartum hemorrhage (PPH), a leading cause of maternal morbidity and mortality globally [10], are at an increased risk of PPD.

Limited data exist to determine whether there is an association between PPH and PPD. Two prior observational studies reported a null association [11,12]. However, these studies did not fully account for confounding bias from pre-existing depression or antidepressant use. In addition, two smaller studies with clinical data have suggested that women may experience severe psychological problems after PPH [13,14]. However, to date, the association between PPH and PPD has not been examined in population-wide data.

We will use population-wide and longitudinal inpatient, outpatient, and pharmacy data from a large unselected population of Swedish women to estimate the association between PPH and PPD, free from the confounding of pre-existing history of depression or exposure to antidepressants.

## Materials and methods

### Ethics approval

In this register-based retrospective study, all data were fully anonymized before being accessed for research purposes. All results were presented on an aggregated level, and no individual could be identified.

Under the Data Protection Act (Swe. lag (2018:218)), no consent to participate was required for analyzing this anonymized data. The Regional Ethical Review Board in Stockholm approved this study (2008/1182-31/4).

### Study population

Our study population comprised 752,988 women with singleton pregnancies who had a live birth at or after 37 weeks and before 42 weeks gestational age between 1 January 2007 and 31 December 2014 in Sweden. For our analysis, we collected data from the Swedish Medical Birth Register (MBR), which contains the International Classification of Diseases, version 10 (ICD-10) codes of diagnoses during pregnancy and childbirth [15]. Using a unique personal identity number [16], we linked data from the Swedish Medical Birth Register to the National Patient Register and the Prescribed Drug Register for diagnoses of depression before and after childbirth. We also linked the data to the Cause of Death Register, the Education Register, and the Total Population Register for information of covariates and censoring [17–19].

**Fig 1:**
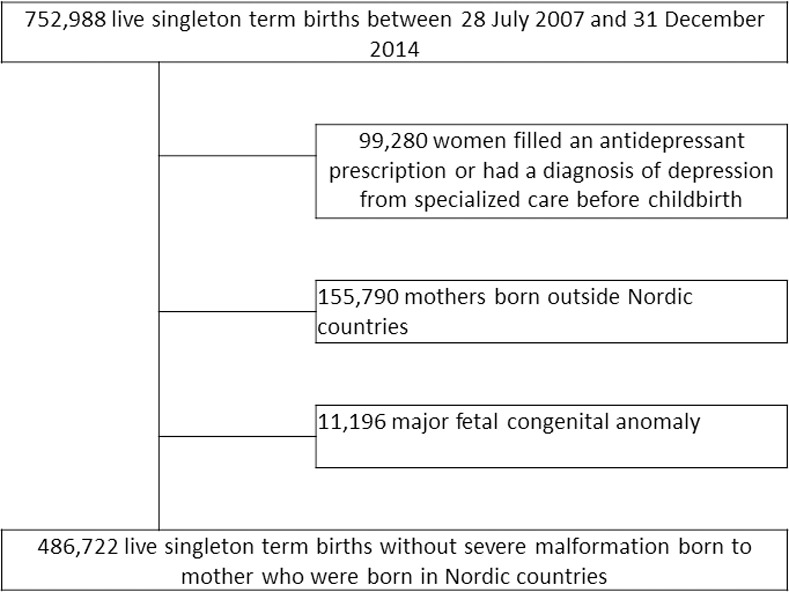
Flowchart of the study population.

We excluded 99,280 women with a history of depression before childbirth, classified as having an ICD-10 diagnosis code for depression (F32-F39) or postpartum depression (F53) from the National Patient Register (starting from 1 January 1973) or a filled prescription for an antidepressant from the Prescribed Drug Register (starting from 1 July 2005). As the birth cohort started in 2007, we obtained at least one year of data of prescribed dispensed drugs before pregnancy to capture information on prenatal depression. Because postpartum depression has been linked to preterm birth or ongoing illness of the baby [20,21], we restricted our study cohort to term births with a non-anomalous pregnancy. To reduce outcome misclassification, we only included women born in Sweden or another Nordic country, who may have fewer language or cultural barriers in seeking specialized care for depression [22]. After the exclusions, there were 486,722 women for analyses (Fig 1). In main analyses, we further excluded women with missing data (N = 38,862, 8.0%) which left 477,860 in the regression models.

### Exposure variable

For our primary exposure of interest, we classified PPH by using an ICD-10 code (O72) during the birth hospitalization. This code was typically applied when the estimated blood loss is over 1000 ml, keeping with guidelines from the Swedish Society of Obstetrics and Gynaecology [23]. Because perioperative bleeding >1000 ml during cesarean birth was coded as hemorrhage (O67.8), we also included this code in our PPH classification. The ICD-10 codes for PPH have been validated in Sweden with high sensitivity (88.9%) and specificity (99.4%) [24].

### Outcome variable

We classified PPD based on the presence of at least one of the following up to 12 months after birth: a filled outpatient prescription for an antidepressant or an ICD-10 code for depression (F32-F39) or postpartum depression (F53) associated with an inpatient hospitalization or outpatient care. Antidepressants were identified using the World Health Organization Anatomical Therapeutic Chemical (ATC) Classification codes beginning with N06A [25]. S1 Table lists all the associated ATC codes of antidepressants prescribed and dispensed at pharmacies under the categories of tricyclic antidepressants, selective serotonin reuptake inhibitors, monoamine oxidase inhibitors, reversible Inhibitor of monoamine oxidase-A, and other antidepressants.

Women were censored if they died or emigrated within 12 months of hospital discharge. Unless PPD was captured before the death, women who committed suicide were censored in the same way as other causes of death. The dates of death and emigration were collected from the Cause of Death Registry and the Total Population Registry, respectively.

### Covariates

Based on literature review [3,26–28] and our expert opinion regarding clinical plausibility, the following covariates were selected as potential confounders: maternal age, parity, highest maternal education, cohabitation status (measured as "cohabiting" or "not-cohabiting" with a partner), maternal smoking status in early pregnancy (categorized into "no smoking", "one to nine cigarettes per day", or "ten or more cigarettes per day"), body mass index (BMI) recorded at the first antenatal visit, gestational age at birth, and birthweight. Data for all covariates (except education) were sourced from the Medical Birth Register. Data for each mother’s highest achieved education level were sourced from the Education Register.

### Missing data

We identified 38,862 (8.0%) women with missing data for at least one covariate. Data for BMI, cohabitation status, and smoking were missing in 6.5%, 4.3%, and 3.9% of the study population. Mode of delivery, birthweight, and maternal age were missing in less than 0.2% of the study population. We considered these variables missing at random and performed complete case analyses for women who did not have missing data for any covariate. S2 Table presents the distribution of all variables from the complete case analysis and those excluded for missingness.

### Statistical analysis

We used Cox proportional-hazards models to examine the association between PPH and PPD, adjusting for maternal and obstetric covariates. Kaplan-Meier survival functions were plotted to describe the association between PPH and PPD up to one year after giving birth. To explore whether the associations between PPH and PPD differ according to the mode of delivery, we performed stratified analyses within each of the following subpopulations: spontaneous vaginal birth, instrumental vaginal birth, planned cesarean birth, and emergency cesarean birth. To account for the potential effect of missing data, in secondary analysis, we performed multiple imputations using chained equations (MICE) with 50 imputations and replicated the main analysis on the complete population. Data management was performed with SAS^®^ version 9.4 (SAS Institute Inc, Cary, NC). All statistical analysis was performed with STATA^®^ version IC 16 (StataCorp, College Station, TX).

### Sensitivity analysis

First, because the risk of PPD may vary according to the severity of blood loss, we reclassified our outcome as severe PPH. Severe PPH was classified by a transfusion ICD-10 procedure code (DR029 from the Medical Birth Register) associated with a PPH ICD-10 code. For this analysis, severe PPH was compared to no PPH as the reference group. Second, to account for clustering of births of the same woman, we restricted our sample to the first birth of the women in the study cohort.

## Results

Of the 486,722 non-anomalous term pregnancies of Nordic-born women who did not have an indication of depression before childbirth, PPH occurred in 31,663 (6.5%) pregnancies, and PPD was diagnosed after 9231 (1.9%) pregnancies.

Table 1 shows the distribution of the study population and the different risks of having PPH by maternal characteristics. Compared to women without PPH, women with PPH were more likely to be primiparous, of advanced age, university educated, non-smokers, give birth at advanced gestational ages, undergo cesarean birth, and give births to infants with higher birthweights.

**Table 1:** Maternal and obstetric characteristics.

		Total births		Postpartum hemorrhage		No hemorrhage	
		n	Col %	n	Row %	n	Row %
All		486,722	100	31,663	6.51	455,059	93.49
Parity							
	1	212,462	43.65	16169	51.07	196,293	43.14
	2–3	255,653	52.53	14,555	45.97	241,098	52.98
	4+	18,607	3.82	939	2.97	17,668	3.88
Maternal age years							
	11–19	6,540	1.34	300	0.95	6,240	1.37
	20–24	58,285	11.98	3,101	9.79	55,184	12.13
	25–29	141,604	29.09	8,693	27.45	132,911	29.21
	30–34	175,384	36.03	11,723	37.02	163,661	35.96
	≥35	104,908	21.55	7,846	24.78	97,062	21.33
	Missing	1				1	
Maternal education							
	Compulsory school ≤9 years	24,536	5.04	1,355	4.28	23,181	5.09
	Secondary school	31,159	6.4	1,933	6.1	29,226	6.42
	University < 3 years	206,716	42.47	13,279	41.94	193,437	42.51
	University ≥ 3 years	224,311	46.09	15,096	47.68	209,215	45.98
Family situation							
	Co-habiting	445,323	91.49	29,066	91.8	416,257	91.47
	Not Co-habiting	20,302	4.17	1,237	3.91	19,065	4.19
	Missing	21,097	4.33	1,360	4.3	19,737	4.34
Maternal smoking status cigarettes/day							
	No smoking	443,046	91.03	29,196	92.21	413,850	90.94
	1–9	19,458	4	995	3.14	18,463	4.06
	≥10	4,992	1.03	255	0.81	4,737	1.04
	Missing	19,226	3.95	1,217	3.84	18,009	3.96
Maternal BMI kg/m2							
	Underweight <18.5	9,367	1.92	542	1.71	8,825	1.94
	Normal weight 18.5 to 24	284,311	58.41	17,719	55.96	266,592	58.58
	Overweight 25 to 29	110,286	22.66	7,592	23.98	102,694	22.57
	Obese Class I 30 to 34	35,716	7.34	2,552	8.06	33,164	7.29
	Obese Class II ≥35	15,200	3.12	1,151	3.64	14,049	3.09
	Missing	31,842	6.54	2,107	6.65	29,735	6.53
Gestational age in weeks							
	37+0–37+6	23,626	4.85	1,474	4.66	22,152	4.87
	38+0–38+6	70,934	14.57	4,440	14.02	66,494	14.61
	39+0–39+6	128,158	26.33	7,005	22.12	121,153	26.62
	40+0–40+6	159,127	32.69	10,237	32.33	148,890	32.72
	41+0–41+6	104,877	21.55	8,507	26.87	96,370	21.18
Mode of delivery							
	Spontaneous vaginal	381,809	78.44	20,386	64.38	361,423	79.42
	Instrumental vaginal	34,809	7.15	3,334	10.53	31,475	6.92
	Planned caesarean section	37,672	7.74	3,647	11.52	34,025	7.48
	Emergency caesarean section	31,667	6.51	4,218	13.32	27,449	6.03
	Missing	765	0.16	78	0.25	687	0.15
Mean birthweight grams [standard deviation]		3,606	[475]	3,752	[505]	3,596	[471]

**Fig 2:**
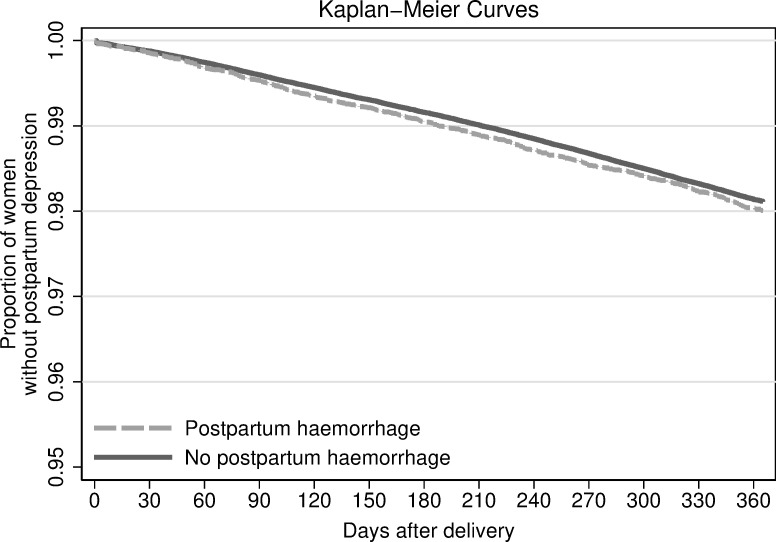
Kaplan-Meier curves for postpartum depression by postpartum hemorrhage exposure status and by mode of delivery.

Over the 12-month postpartum period, the risk of PPD was 2.0% (630/31,663) among women with PPH and 1.9% (8601/455,059) among women who did not experience PPH. Fig 2 shows the Kaplan-Meier survival curves for PPD by PPH exposure status in the study population. Log-rank test suggested no difference between the PPH exposed and unexposed groups (p-value = 0.20). The unadjusted Cox proportional hazard model showed no significant association between PPH and PPD (hazard ratio (HR) = 1.06, 95% CI 0.97, 1.15). After adjusting for maternal age, parity, education, cohabitation status, maternal smoking status, and early pregnancy maternal BMI, gestational age, and birthweight, the null association remained between PPH and PPD (adjusted HR 1.08, 95% CI 0.99, 1.17). In the multiple imputation models accounting for missing data, we still observed a null association between PPH and PPD (adjusted HR 1.07, 95% CI 0.99, 1.16) (S3 Table).

Table 2 shows the results when we stratified our cohort according to mode of delivery. We observed null associations between PPH and PPD across all strata: spontaneous vaginal birth (adjusted HR 1.03, 95% CI 0.93, 1.16), instrumental vaginal birth (adjusted HR 1.22, 95% CI 0.92, 1.61), planned cesarean birth (adjusted HR 1.09, 95% CI 0.88, 1.36) and unplanned cesarean birth (adjusted HR 1.12, 95% CI 0.90, 1.39).

**Table 2:** Adjusted Hazard Ratios for postpartum depression after exposure to postpartum hemorrhage (PPH).

	Non-instrumental vaginal birth N = 352,185			Instrumental vaginal birth N = 31,998			Planned caesarean birth N = 34,525			Unplanned caesarean birth N = 29,152		
	n/N	%	HR (95%CI)	n/N	%	HR (95%CI)	n/N	%	HR (95%CI)	n/N	%	HR (95%CI)
No PPH	6067/333,359	1.82	1.00 (Reference)	459/28,922	1.59	1.00 (Reference)	785/31,226	2.51	1.00 (Reference)	577/25,268	2.28	1.00 (Reference)
PPH	334/18,826	1.77	1.03 (0.93, 1.16)	56/3076	1.82	1.22 (0.92, 1.61)	90/3299	2.73	1.09 (0.88, 1.36)	99/3884	2.55	1.12 (0.90, 1.39)

Adjusted for maternal age, family situation, education, parity, gestational age, birthweight, maternal smoking status, and early pregnancy maternal BMI.

Abbreviations: HR, hazard ratio; CI, confidence interval; PPH, postpartum hemorrhage.

### Sensitivity analysis

In our first sensitivity analysis in which our outcome was severe PPH, a null association was observed between severe PPH (PPH with transfusion) and PPD (adjusted HR: 0.99, 95% CI 0.75, 1.29). Table 3 shows the association between severe PPH and PPD stratified by mode of delivery. Consistent with the main finding, there was no increased risk of PPD associated with severe PPH across all strata. The associations between PPH without transfusion and PPD were consistent with the main analyses, with the point estimation being the highest in instrumental vaginal birth but the confidence intervals overlapping null 1.25 (0.93, 1.67). In the second sensitivity analysis restricting the study cohort to the first deliveries only, a null association was also observed overall (adjusted HR = 1.08, 95% CI 0.98, 1.19) and across the strata by mode of delivery (S4 Table).

**Table 3:** Adjusted Hazard Ratios for postpartum depression after exposure to postpartum hemorrhage classified by with and without blood transfusion.

	Non-instrumental vaginal birth N = 352,185			Instrumental vaginal birth N = 31,998			Planned caesarean birth N = 34,525			Unplanned caesarean birth N = 29,152		
	n/N	%	HR (95%CI)	n/N	%	HR (95%CI)	n/N	%	HR (95%CI)	n/N	%	HR (95%CI)
No PPH	6067/333,359	1.82	1.00 (Reference)	459/28,922	1.59	1.00 (Reference)	785/31,226	2.51	1.00 (Reference)	577/25,268	2.28	1.00 (Reference)
PPH without transfusion	298/16,874	1.77	1.03 (0.92, 1.16)	50/2689	1.86	1.25 (0.93, 1.67)	86/3063	2.81	1.13 (0.91, 1.42)	92/3522	2.61	1.15 (0.92, 1.44)
PPH with transfusion	36/1952	1.84	1.09 (0.78, 1.51)	6/387	1.55	1.02 (0.45, 2.28)	4/236	1.69	0.63 (0.24, 1.69)	7/362	1.93	0.83 (0.39, 1.76)

Adjusted for maternal age, family situation, education, parity, gestational age, birthweight, maternal smoking status, and early pregnancy maternal BMI.

Abbreviations: HR, hazard ratio; CI, confidence interval; PPH, postpartum hemorrhage.

## Discussion

### Principal findings

In this population-wide study of 486,722 term pregnancies in Sweden, we observed that PPH was not associated with PPD. Results of our sensitivity and stratified analyses were similar to those of our main analysis, which provide additional evidence to substantiate the robustness of our main findings.

### Study strengths

A key strength is that the large size of a national sample of all women who gave birth in Sweden between 2007 and 2014. Linked data from Swedish datasets also allowed us to follow up for 12 months after giving birth and account for a detailed set of pre-pregnancy and pregnancy-related confounders, which is clinically relevant and adding to previous knowledge. In addition, our dataset did not contain any missing data for our primary exposure (PPH) or outcome (PPD).

### Study limitations

Because the accuracy of PPD diagnoses using ICD-10 codes in Sweden is unknown, it is unclear whether PPD was over- or under-ascertained. However, in Sweden, universal screening for PPD with the Edinburgh Postnatal Depression Scale (EPDS) [29] is provided to mothers at 6–8 weeks postpartum during child health service [30]. To confirm our findings, future studies may use EPDS data to assess the accuracy of PPD diagnostic coding with ICD-10 codes.

We attempted to account for depression that existed before childbirth. But we could not identify women with antepartum depression that did not have an ICD-10 code for depression and did not receive antidepressants during pregnancy. Therefore, some PPD cases may have had an antepartum depression, and the observed association was confounded by pre-existing depression. Also, excluding women with a history of depression limited the generalizability of our findings.

Besides depressive symptoms, PPH may also induce emotional trauma and posttraumatic stress disorder [31]. However, few studies have investigated this link [32]. Future population-based studies may need to examine if PPH increases risks of other psychiatric morbidities such as posttraumatic stress disorder or anxiety.

Although the accuracy of ICD-10 coding for PPH is high [24], validation studies rely on blood loss measurements for determining PPH coding accuracy. It is well recognized that blood loss estimation can be inaccurate, with blood loss often being underestimated in severe cases [33] and in caesarean births [34]. Therefore, the prevalence of PPH may have been underestimated, which may have biased point estimates in our regression analyses toward the null. Of note, the PPH diagnosis was given at >1000 ml estimated blood loss in Sweden during the study period, which is higher than the commonly used threshold of 500 ml [35]. Our null finding with the higher threshold also suggested a lack of substantial impact of PPH on PPD. Besides PPH, postpartum anemia could not be accurately measured given the data, leaving the association between postpartum anemia and PPD unclear. Thus, future studies with appropriate data may help elucidate the link between postpartum anemia and PPD.

To avoid confounding by indication, we excluded women with a known history of depression. This likely explains why the PPD prevalence was lower than the PPD prevalence reported in overall birthing populations [3].

### Interpretation

Based on our findings, PPH was not associated with an increased risk of PPD. In particular, whether PPH was associated with increased risk of PPD after instrumental vaginal birth remains inconclusive. These findings require validation in large scale prospective studies with a more accurate measure of PPD. If confirmed, women who experience PPH may not require additional surveillance for PPD after childbirth.

Compared to our findings, previous studies examining the association between PPH and PPD produced inconsistent results. This may be partly because these studies did not account for confounding from pre-existing depression or antidepressant exposure [11–14]. Furthermore, definitions for PPH and PPD vary across these studies. A qualitative study by Sentilhes et al. suggested that women who had a PPH requiring arterial embolization had long-term psychological trauma, including anxiety and depression [13]. In contrast, an Australian clinical based cohort study on women who had PPH of over 1500 ml or a low postpartum hemoglobin concentration [14] concluded that the physical and emotional well-being of the cohort did not differ from women in a general postpartum population, mirroring the findings of our study. Similarly, a Swedish population-based survey examining 446 women from Uppsala county [11] showed no association between PPH over 1000 ml and PPD, defined as having the EPDS score ≥12.

Compared to these previous studies, our study used a large sample from the national population. We deliberately focused on Nordic-born women who were more similar in accessing medical care for PPD, who had no pre-existing depression, and who gave term births. By restricting on the confounders and factors associated with measurement errors, we were better able to disentangle the association between PPH and PPD risk.

Other pre-pregnancy, antepartum, and postpartum social and psychosocial factors may be more predictive for PPD, in contrast to a single risk factor of PPH. Future studies are warranted to explore the interplay of obstetrical, social, and psychosocial factors in affecting PPD risk. It is also unclear whether a similar association would be observed in countries with a differing prevalence of PPH or PPD, in addition to different social, psychosocial factors, and obstetric practices. Therefore, future studies are needed to find out if the lack of a strong association between PPD and PPD is generalizable in other countries.

## Conclusions

The findings from this population-based cohort suggest that PPH is not associated with PPD up to 12 months after giving birth among women without a prior history of depression. Future research is needed to validate these findings and focus on women with a history of depression.

## Supporting information

S1 Table List of antidepressants. (DOCX)

S2 Table Distribution of variables by inclusion and exclusion for complete case analysis. (DOCX)

S3 Table Adjusted Hazard Ratios for postpartum depression after exposure to PPH in the whole study population after multiple imputation. (DOCX)

S4 Table Adjusted Hazard Ratios for postpartum depression after exposure to PPH among first delivery in the study period. (DOCX)
