# Supplementary material for: The association between postpartum hemorrhage and postpartum depression: A Swedish national register-based study
Source: PLoS One. 2021 Aug 11;16(8):e0255938. doi: 10.1371/journal.pone.0255938 (PMC8357098; doi:10.1371/journal.pone.0255938)
Supplement: S1 Table — (DOCX) [file pone.0255938.s001.docx]

S1 Table. List of antidepressants

| Category | Substance | ATC |
| --- | --- | --- |
|  |  |  |
| Non-selective MRI | Imipramine | N06AA02 |
|  | Clomipramine | N06AA04 |
|  | Trimipramine | N06AA06 |
|  | Lofepramine | N06AA07 |
|  | Amitriptyline | N06AA09 |
|  | Nortriptyline | N06AA10 |
|  | Maprotiline | N06AA21 |
| SSRI | Fluoxetine | N06AB03 |
|  | Citalopram | N06AB04 |
|  | Paroxetine | N06AB05 |
|  | Sertraline | N06AB06 |
|  | Fluvoxamine | N06AB08 |
|  | Escitalopram | N06AB10 |
| MAOI | Phenelzine | N06AF03 |
|  | Tranylcypromine | N06AF04 |
| RIMA | Moclobemide | N06AG02 |
| Other Antidepressants | Oxitriptan | N06AX01 |
|  | Tryptophan | N06AX02 |
|  | Mianserin | N06AX03 |
|  | Trazodone | N06AX05 |
|  | Nefazodone | N06AX06 |
|  | Mirtazapine | N06AX11 |
|  | Bupropion | N06AX12 |
|  | Tianeptine | N06AX14 |
|  | Venlafaxine | N06AX16 |
|  | Milnacipran | N06AX17 |
|  | Reboxetine | N06AX18 |
|  | Duloxetine | N06AX21 |
|  | Agomelatine | N06AX22 |
|  | Desvenlafaxine | N06AX23 |
|  | Vortioxetine | N06AX26 |
