# Supplementary material for: The association between postpartum hemorrhage and postpartum depression: A Swedish national register-based study
Source: PLoS One. 2021 Aug 11;16(8):e0255938. doi: 10.1371/journal.pone.0255938 (PMC8357098; doi:10.1371/journal.pone.0255938)
Supplement: S2 Table — (DOCX) [file pone.0255938.s002.docx]

S2 Table. Distribution of variables by inclusion and exclusion for complete case analysis

|  |  | Study population | | Included for complete case analysis | | Excluded for missingness | |
| --- | --- | --- | --- | --- | --- | --- | --- |
|  |  | n | Col % | n | Col % | n | Col % |
| All |  | 486,722 | 100 | 447,860 | 100 | 38,862 | 100 |
| Postpartum depression | |  |  |  |  |  |  |
|  | No | 477,491 | 98.1 | 439,393 | 98.11 | 38,098 | 98.03 |
|  | Yes | 9,231 | 1.9 | 8,467 | 1.89 | 764 | 1.97 |
| Postpartum haemorrhage | |  |  |  |  |  |  |
|  | No | 455,059 | 93.49 | 418,775 | 93.51 | 36,284 | 93.37 |
|  | Yes | 31,663 | 6.51 | 29,085 | 6.49 | 2,578 | 6.63 |
| Parity |  |  |  |  |  |  |  |
|  | 1 | 212,462 | 43.65 | 195,663 | 43.69 | 16,799 | 43.23 |
|  | 2-3 | 255,653 | 52.53 | 235,278 | 52.53 | 20,375 | 52.43 |
|  | 4+ | 18,607 | 3.82 | 16,919 | 3.78 | 1,688 | 4.34 |
| Maternal age years | |  |  |  |  |  |  |
|  | 11-19 | 6,540 | 1.34 | 5,925 | 1.32 | 615 | 1.58 |
|  | 20-24 | 58,285 | 11.98 | 53,984 | 12.05 | 4,301 | 11.07 |
|  | 25-29 | 141,604 | 29.09 | 130,967 | 29.24 | 10,637 | 27.37 |
|  | 30-34 | 175,384 | 36.03 | 161,195 | 35.99 | 14,189 | 36.51 |
|  | ≥35 | 104,908 | 21.55 | 95,789 | 21.39 | 9,119 | 23.47 |
|  | Missing | 1 | 0 | 0 | 0 | 1 | 0 |
| Maternal education | |  |  |  |  |  |  |
|  | Compulsory school ≤9 years | 24,536 | 5.04 | 22,398 | 5 | 2,138 | 5.5 |
|  | Secondary school | 31,159 | 6.4 | 28,562 | 6.38 | 2,597 | 6.68 |
|  | University < 3 years | 206,716 | 42.47 | 190,589 | 42.56 | 16,127 | 41.5 |
|  | University ≥ 3 years | 224,311 | 46.09 | 206,311 | 46.07 | 18,000 | 46.32 |
| Family situation | |  |  |  |  |  |  |
|  | Co-habiting | 445,323 | 91.49 | 428,809 | 95.75 | 16,514 | 42.49 |
|  | Not Co-habiting | 20,302 | 4.17 | 19,051 | 4.25 | 1,251 | 3.22 |
|  | Missing | 21,097 | 4.33 | 0 | 0 | 21,097 | 54.29 |
| Maternal smoking status cigarettes / day | | |  |  |  |  |  |
|  | No smoking | 443,046 | 91.03 | 424,523 | 94.79 | 18,523 | 47.66 |
|  | 1-9 | 19,458 | 4 | 18,581 | 4.15 | 877 | 2.26 |
|  | ≥10 | 4,992 | 1.03 | 4,756 | 1.06 | 236 | 0.61 |
|  | Missing | 19,226 | 3.95 | 0 | 0 | 19,226 | 49.47 |
| Maternal BMI kg/m2 | |  |  |  |  |  |  |
|  | Underweight <18.5 | 9,367 | 1.92 | 9,230 | 2.06 | 137 | 0.35 |
|  | Normal weight 18.5 to 24 | 284,311 | 58.41 | 280,061 | 62.53 | 4,250 | 10.94 |
|  | Overweight 25 to 29 | 110,286 | 22.66 | 108,555 | 24.24 | 1,731 | 4.45 |
|  | Obese Class I 30 to 34 | 35,716 | 7.34 | 35,097 | 7.84 | 619 | 1.59 |
|  | Obese Class II ≥35 | 15,200 | 3.12 | 14,917 | 3.33 | 283 | 0.73 |
|  | Missing | 31,842 | 6.54 | 0 | 0 | 31,842 | 81.94 |
| Gestational age in weeks | |  |  |  |  |  |  |
|  | 37+0--37+6 | 23,626 | 4.85 | 21,424 | 4.78 | 2,202 | 5.67 |
|  | 38+0--38+6 | 70,934 | 14.57 | 64,709 | 14.45 | 6,225 | 16.02 |
|  | 39+0--39+6 | 128,158 | 26.33 | 117,921 | 26.33 | 10,237 | 26.34 |
|  | 40+0--40+6 | 159,127 | 32.69 | 146,887 | 32.8 | 12,240 | 31.5 |
|  | 41+0--41+6 | 104,877 | 21.55 | 96,919 | 21.64 | 7,958 | 20.48 |
| Mode of delivery | |  |  |  |  |  |  |
|  | Spontaneous vaginal | 381,809 | 78.44 | 352,185 | 78.64 | 29,624 | 76.23 |
|  | Instrumental vaginal | 34,809 | 7.15 | 31,998 | 7.14 | 2,811 | 7.23 |
|  | Planned caesarean section | 37,672 | 7.74 | 34,525 | 7.71 | 3,147 | 8.1 |
|  | Emergency caesarean section | 31,667 | 6.51 | 29,152 | 6.51 | 2,515 | 6.47 |
|  | Missing | 765 | 0.16 | 0 | 0 | 765 | 1.97 |
| Mean birthweight grams | |  |  |  |  |  |  |
|  | Missing | 587 | 0.12 | 0 | 0 | 587 | 1.51 |
