# Supplementary material for: The association between postpartum hemorrhage and postpartum depression: A Swedish national register-based study
Source: PLoS One. 2021 Aug 11;16(8):e0255938. doi: 10.1371/journal.pone.0255938 (PMC8357098; doi:10.1371/journal.pone.0255938)
Supplement: S3 Table — (DOCX) [file pone.0255938.s003.docx]

S3 Table. Adjusted Hazard Ratios for postpartum depression after exposure to PPH in the whole study population after multiple imputation

|  | Non-instrumental vaginal delivery N=1,890,303 | | | Instrumental delivery N=177,547 | | | Planned caesarean delivery N=200,918 | | | Unplanned caesarean delivery N=159,991 | | |
| --- | --- | --- | --- | --- | --- | --- | --- | --- | --- | --- | --- | --- |
|  | n/N | % | HR (95%CI) | n/N | % | HR (95%CI) | n/N | % | HR (95%CI) | n/N | % | HR (95%CI) |
| No PPH | 33,684/1,789,608 | 1.88 | 1.00 (Reference) | 2739/161,131 | 1.70 | 1.00 (Reference) | 5351/179,194 | 2.99 | 1.00 (Reference) | 2461/138,639 | 1.78 | 1.00 (Reference) |
| PPH | 1949/100,695 | 1.94 | 1.03 (0.93, 1.15) | 268/16,529 | 1.62 | 1.19 (0.91, 1.56) | 480/21,838 | 2.20 | 1.05 (0.85, 1.29) | 480/21,423 | 2.24 | 1.13 (0.91, 1.39) |

Adjusted for maternal age, family situation, education, parity, gestational age, birthweight, maternal smoking status, early pregnancy maternal BMI

Abbreviations: HR, hazard ratio; CI, confidence interval; PPH, postpartum haemorrhage
