# Supplementary material for: The association between postpartum hemorrhage and postpartum depression: A Swedish national register-based study
Source: PLoS One. 2021 Aug 11;16(8):e0255938. doi: 10.1371/journal.pone.0255938 (PMC8357098; doi:10.1371/journal.pone.0255938)
Supplement: S4 Table — (DOCX) [file pone.0255938.s004.docx]

S4 Table. Adjusted Hazard Ratios for postpartum depression after exposure to PPH among first delivery in the study period

|  | Non-instrumental vaginal delivery N=254,259 | | | Instrumental delivery N=29,563 | | | Planned caesarean delivery N=25,515 | | | Unplanned caesarean delivery N=25,388 | | |
| --- | --- | --- | --- | --- | --- | --- | --- | --- | --- | --- | --- | --- |
|  | n/N | % | HR (95%CI) | n/N | % | HR (95%CI) | n/N | % | HR (95%CI) | n/N | % | HR (95%CI) |
|  |  |  |  |  |  |  |  |  |  |  |  |  |
| No PPH | 4200/239,963 | 1.75 | 1.00 (Reference) | 420/26,675 | 1.57 | 1.00 (Reference) | 539/23,003 | 2.34 | 1.00 (Reference) | 495/22,030 | 2.25 | 1.00 (Reference) |
| PPH | 238/14,296 | 1.66 | 1.02 (0.89, 1.16) | 55/2888 | 1.90 | 1.27 (0.96, 1.69) | 66/2512 | 2.63 | 1.13 (0.87, 1.46) | 82/3358 | 2.44 | 1.08 (0.86, 1.39) |

Adjusted for maternal age, family situation, education, parity, gestational age, birthweight, maternal smoking status, early pregnancy maternal BMI

Abbreviations: HR, hazard ratio; CI, confidence interval; PPH, postpartum haemorrhage
